# Exploring health related quality of life for women with breast cancer in Ireland and Québec, Canada throughout the COVID-19 pandemic

**DOI:** 10.1038/s41598-024-84852-9

**Published:** 2025-02-01

**Authors:** Charlotte Myers, Kathleen Bennett, Caitriona Cahir, Josée Savard, Sophie Lauzier

**Affiliations:** 1 School of Population Health, RCSI University of Medicine and Health Sciences, Dublin, Ireland; 2 Data Science Centre, School of Population Health, RCSI University of Medicine and Health Sciences, Dublin, Ireland; 3 Faculty of Social Sciences, CHU de Québec-Université Laval Research Center, Cancer Research Center, Université Laval, Québec, Canada; 4 Faculty of Pharmacy, CHU de Québec-Université Laval Research Center, Cancer Research Center, Université Laval, Québec, Canada

**Keywords:** COVID-19, Breast cancer, Psychosocial stressors, Quality of life, Cross-country comparison, Psycho-oncology, Cancer, Health care, Oncology

## Abstract

The long-term consequences from the COVID-19 pandemic on breast cancer (BC) is highly unknown, however persisting unmet needs and psychosocial difficulties are likely. The objectives of this study were to evaluate the change in health-related quality of life (HR-QoL) from the pandemic to post-pandemic for women living with a diagnosis of BC and to assess the association between COVID-19 stressor impact and HR-QoL in Ireland and Québec, Canada. Women with a diagnosis of BC were initially enrolled in the cohort study. HR-QoL was assessed during the pandemic (2020–2021) and post-pandemic periods (2022). COVID-19 stressor impact was computed post-pandemic, and change in HR-QoL during and post-pandemic was compared between Ireland and Québec using independent t-tests. Multivariable analysis of covariance (ANCOVA) was used to evaluate the association between COVID-19 stressor impact and changes in HR-QoL, and compare it between Ireland and Québec. 405 participants were included from both settings (Ireland *n* = 267; Québec *n* = 138). The average HR-QoL improved from the COVID-19 pandemic to post-pandemic, and there were no differences between Ireland and Québec. Women with high COVID-19 stressor impact (18.9% of participants) had a significantly smaller improvement in their overall HR-QoL compared to those with low COVID-19 stressor impact, and this was evident in Ireland (*p* < 0.004) and Québec (*p* < 0.0001) but there were no significant differences between Ireland and Québec (interaction p-value > 0.05). Overall, HR-QoL for women with BC improved from pandemic to post-pandemic period. However, similarly in both settings, women who experienced higher levels of COVID-19-related stress had a slower recovery in HR-QoL. These results can guide decisions about health services and policies to adequately address the on-going effect of the pandemic and also prepare for future health crises.

**Supplementary Information:**

The online version contains supplementary material available at 10.1038/s41598-024-84852-9.

## Introduction 

Globally, the COVID-19 pandemic has impacted breast cancer (BC) services since the World Health Organisation (WHO) declared it a global pandemic in March, 2020. Women living with a diagnosis of BC have experienced disruptions across the cancer care continuum, including screening services, active treatment, routine care, and supportive care services^1^. Short-term consequences from such disruptions include clinical repercussions, such as fewer diagnoses of early-stage BC^2^, and psychosocial repercussions, such as diminished quality of life (QoL)^3^. The long-term consequences from the COVID-19 pandemic on BC is highly unknown, however it is predicted that the morbidity and mortality rates may increase in coming years^4^ and it is likely women will continue to experience psychosocial difficulties^5,6^. There is a need to conduct longitudinal studies to quantify long-term consequences and guide decisions about health services and policies to adequately address on-going unmet needs. Results from such studies can also help health systems prepare for future health crises.

Countries worldwide approached the COVID-19 pandemic differently, through various guidelines and restrictions given political and social reasoning^7^. Most countries implemented public and social measures in response to the pandemic to resist the spread of disease and protect the health of the public. These measures typically included restrictions on mobility, social distancing, self-isolation, the use of face-masks, and vaccination plans^8,9^. In addition, while many countries have published country specific research on the impact of COVID-19 on BC, which most includes the impact on BC services and health-related outcomes^1^, there is limited evidence comparing the impact of COVID-19 on health outcomes across countries and how this may have differed. In order to compare the impact of COVID-19 across countries, similar measures of data collection and analyses need to be adopted^10,11^.

Ireland, a member state of the European Union (EU), operates on a complex public-private health system, where public health care is largely tax-funded, yet the majority of citizens have to contribute to the payment of their health services. This dual health care system creates inequalities for health services^12^, including cancer^13^. During the COVID-19 pandemic, Ireland experienced longer and more severe restrictions compared to other countries globally^7^. Regarding cancer care, health services, especially screening programmes, were disrupted given governmental restrictions and prioritisation to COVID-19 cases and other urgent health issues^14^. In comparison, Québec, Canada operates primarily with covercare, which is publicly funded and under the responsibility of the provincial governments^15^. Québec implemented a series of measures that have varied according to the phases of the pandemic, from the closure of public places and social restrictions during the first wave of the pandemic to establishing a curfew in January 2021^16^. Regarding cancer care, symptomatic cancer cases were prioritised and the disruption to screening services impacted the number of cancer diagnoses^17,18^.

The COVID-19 pandemic has highlighted the importance of health system resilience, which varies given certain political, social, and cultural contexts^19^. The social determinants of health (SDH) framework outlines the interplay of diverse factors that impact health, including macro-level determinants, such as political systems, social structures, and health policies, and micro-level determinants, such as psychosocial stressors, socio-economic status (SES), and race/ethnicity^20,21^. The SDH framework has been applied to COVID-19 in many capacities^22^, however, there is limited research analysing country as a macro-level determinant, and psychosocial stressors specific to COVID-19 as micro-level determinants for health and well-being.

The aim of this study is to determine the association between COVID-19 stressors and changes in health-related QoL (HR-QoL) from the pandemic to post-pandemic for women living with a diagnosis of BC in Ireland compared to Québec, Canada. The specific research objectives are:

1. To estimate and compare the prevalence of COVID-19 stressors;
2. To compare changes in HR-QoL and specific HR-QoL subscales from the pandemic to post-pandemic;
3. To explore differences in the association between COVID-19 stressors and changes in HR-QoL for women in Ireland compared to Québec.

## Methods

The STROBE guidelines were utilised for reporting the observational study^23^.

### Study design

The overall research study design was conducted as a two-phase prospective mixed-methods cohort study, including a first timepoint (T1—referred in this article as the pandemic period) which corresponds with government restrictions in response to the COVID-19 pandemic in both Ireland (September 2020 to March 2021) and Québec (November 2020 to March 2021), and a second timepoint (T2—referred as the post-pandemic period) which corresponds to post-restrictions starting in June 2022 for both countries, and lasting until August 2022 (Ireland) and September 2022 (Québec). Both settings used similar eligibility criteria and standardised measures, and the data were collected at similar timepoints. All methods were performed in accordance to relevant guidelines and regulations as specified through ethical approval at both study sites.

### Participants

Women with a diagnosis of BC from both Ireland and Québec were initially recruited for the study cohort by an online campaign, involving social media and affiliated mailing lists starting in September, 2020. Women were eligible to take part in the cohort study if they had a diagnosis of BC within the last 5 years, were living in Ireland or Québec, were over 18 years of age, and were English speaking (Ireland) or French speaking (Québec). With initial eligibility, women were prompted to complete an online survey. Women were asked to complete a questionnaire at both timepoints, T1 and T2, to understand their experience during the COVID-19 pandemic as well as during post-pandemic.

Additionally in Ireland, paper-based surveys were distributed to women receiving BC services from two cancer centres via non-probability sampling techniques to increase the representativity of the sampling for initial recruitment into the baseline timepoint. At T1, 162 participants completed the online survey, and 225 participants completed the survey through their BC clinic, and women were recruited into the baseline cohort of the study during the same time period^24^.

### Outcome measures

The Functional Assessment of Cancer Therapy- Breast (FACT-B) was used to assess HR-QoL at T1 and T2. This measure includes the Functional Assessment of Cancer Therapy- General (FACT-G) which assesses overall well-being specific to cancer, including physical well-being (PWB); social well-being (SWB); emotional well-being (EWB); and functional well-being (FWB). In addition to these subscales, the FACT-B includes an additional BC sub-scale (BCS), including 10 items which measures concerns specific to BC. The FACT-B includes 37 items using a 5-point Likert scale ranging from 0 = “not at all” to 4 = “very much.”^25^ A score can be calculated for each subscale (PWB score range: 0–28; SWB score range: 0–28; EWB score range: 0–24; FWB score range: 0–28; and BCS score range: 0–40) as well as an overall score for the FACT-G (score range: 0–108) and for the FACT-B (score range: 0–148). A higher score indicates better HR-QoL.

### COVID-19 stressor impact

The impact of COVID-19 was assessed using an adaptation of the COVID-19 Stressors Questionnaire at T2^26^. This questionnaire developed at the start of the pandemic contained originally 14 items and 2 items were added as our understanding of the pandemic impact was evolving. This adapted questionnaire includes 16 questions on daily life, life events, workplace, financial stability, treatment changes, and health-related concerns specific to BC and COVID-19 that were experienced during the pandemic. The full questionnaire can be found in the *Supplementary 1.* Participants were asked if they had experienced the stressor and to what extent the stressor caused worry or concern, ranging from 0= “not at all” to 4= “extremely.” When a participant did not experience the stressor, a value of 0 was attributed for the extent of worry or concern related to this stressor. The average of the 16 items was calculated and participants with an average level of concern below 1 were categorized as no/low COVID-19 stressor impact and participants with an average level of concern above or equal to 1 were categorized as high COVID-19 stressor impact.

### Potential socio-demographic and clinical confounders

Potential socio-demographic confounders included age, region, education level, employment status, relationship status, and income at T2 to reflect the accurate and timely demographic data. Age remained a continuous variable, while all other demographic variables were organised categorically. For region, participants were categorised as urban (e.g. living in a city) vs. rural (e.g. living in a town/village/ countryside). For education level, participants were categorised as low (primary/secondary) education vs. high (completed university degree and above) education. For employment status, participants were categorized as employed (full-time and/ or part-time) and not employed (unable to work due to sickness/disability, redundant, retired, or looking after family and home). For relationship status, participants were categorised as either married/in a long-term relationship or not in a relationship (single, separated, divorced, and/or widowed). For income, participants were categorised as high income (above 40,000 euro / 60,000 Canadian dollar per annum) vs. lower income (below 40,000 euro / 60, 000 Canadian dollar per annum) based on census data^27,28^.

Potential clinical confounders included most recent BC diagnosis stage, co-morbidities, and time since most recent BC diagnosis at T2. For most recent BC diagnosis, participants were categorised with a non-metastatic (stage I-III) diagnosis vs. a metastatic (stage IV) diagnosis. For co-morbidities, participants were categorised as having one or no co-morbidities or two or more co-morbidities in addition to BC. For time since most recent BC diagnosis, participants were categorised as being diagnosed between 2019 and 2022 or 2015–2018 to reflect potential diagnosis and treatment in relation to COVID-19 restrictions.

### Statistical analysis

Descriptive statistics on COVID-19 stressor impact, socio-demographic, and clinical confounders included means and standard deviations (SD) for continuous data and frequencies and percentages for categorical data were calculated. The prevalence of each COVID-19 stressor was established by country. The extent of worry or concern caused by each stressor was assessed using medians and interquartile ranges (IQR) in each country for participants who reported having experienced the stressor. Differences between COVID-19 stressors, socio-demographic, and clinical potential confounders were compared across the two countries using chi-squared tests for categorical data and t-tests for continuous data. Differences in mean between T1 and T2 for HR-QoL per country are reported using paired t-tests. Differences in HR-QoL between the two countries at both T1 and T2 timepoints and the change score (T2-T1) were assessed using independent t-tests. Multivariable analysis of covariance (ANCOVA) was used to assess the effect of COVID-19 stressor impact on each post-pandemic HR-QoL subscales, using pandemic HR-QoL (T1) as the covariate of the ANCOVA, and adjusting for potential socio-demographic and clinical confounders. Additional interaction terms with country for the potential confounders were tested for significance using a hierarchical approach, in which potential confounders were strategically entered into the models in stages to test statistical significance^29^. Interaction terms statistically significant for at least one HR-QoL outcome (*p* < 0.05) were included in all models for better adjustment by country. This included education, income, and co-morbidity status. All other potential confounders (e.g. age, region, employment status, relationship status, cancer stage, and time since diagnosis) were included in the model without interaction terms. P-values < 0.05 were recognised as statistically significant. Additional details can be found in *Supplementary 2*. Any missing data were omitted from analyses and the data were analysed using Stata Version 16.1 (StataCorp, College Station, TX, USA) and SAS 9.4 (SAS Institute Inc., Cary, NC, USA).

## Results

### Patient characteristics

In total, 405 women, including 267 participants from Ireland (65.9%) and 138 participants from Québec (34.1%), were included in the study and completed T1 and T2 measures. The average age for participants in Ireland was 56.3 years (SD = 10.1) and the average age for participants in Québec was 55.3 years (SD = 11.4) (*p* = 0.35). The majority of participants were employed (50.9% for Ireland vs. 57.3% for Québec), were married or in a long-term relationship (73.5% for Ireland vs. 71% for Québec), reported a non-metastatic BC diagnosis (90.1 for Ireland vs. 91% for Québec), reported 1 or less co-morbidities in addition to BC (76.0% for Ireland vs. 74.6% for Québec ), and were diagnosed within the last 3 years (56.1% for Ireland vs. 58.4% for Québec). These differences between Ireland and Québec were not statistically significant. A significantly higher percentage of participants from Ireland, compared to Québec, reside in rural regions (58.4% for Ireland vs. 16.5% for Québec, *p* < 0.001) and reported an education level at or above a university degree (70.6% for Ireland vs. 51.5% for Québec, *p* < 0.001). In addition, a significantly higher percentage of participants from Québec reported a high annual household income (78.3%) compared to Ireland (61.4%) (*p* = 0.001) (Table 1).

**Table 1:** Participant characteristics and COVID-19 stressor impact level (*n* = 405).

	Ireland (*n* = 267) N (%)	Québec, Canada (*n* = 138) N (%)	*p*-value
COVID-19 stressor impact			
Low impact	225 (84.9)	102 (73.9)	0.007*
High impact	40 (15.1)	36 (26.1)	
Age- mean (SD)	56.3 (10.1)	55.3 (11.4)	0.347
Region			
Urban	109 (41.6)	101 (83.5)	< 0.001*
Rural	153 (58.4)	20 (16.5)	
Education			
High	175 (70.6)	71 (51.5)	< 0.001*
Low	73 (29.4)	67 (48.5)	
Employment			
Employed	135 (50.9)	79 (57.3)	0.229
Not employed	130 (49.1)	59 (42.8)	
Relationship status			
Married/ in a relationship	194 (73.5)	98 (71.0)	0.598
Not in a relationship	70 (26.5)	40 (29.0)	
Income			
High	156 (61.4)	94 (78.3)	0.001*
Low	98 (38.6)	26 (21.7)	
Cancer stage (most recent)			
Non-metastatic	227 (90.1)	127 (92.0)	0.525
Metastatic	25 (9.9)	11 (8.0)	
Co-morbidities			
1 or less	203 (76.0)	103 (74.6)	0.757
2 or more	64 (24.0)	35 (25.4)	
Time since most recent cancer diagnosis			
2015–2018	107 (43.9)	57 (41.6)	0.671
2019–2022	137 (56.1)	80 (58.4)	

### COVID-19 stressors and impact

Overall, 18.9% (*n* = 76) of participants from both countries reported a high COVID-19 stressor impact. A significantly higher percentage (*n* = 36, 26.1%) of participants from Québec reported a high COVID-19 stressor impact compared to Ireland (*n* = 40, 15.1%) (*p* = 0.007) (Table 1). *Supplementary 1* details each COVID-19 stressor and compares its prevalence between Ireland and Québec, as well as the extent of concern or worry it caused among women who experienced this specific stressor. Participants from Québec reported the following COVID-19 stressors significantly more frequently than participants from Ireland: having a loved one become ill following probable or certain exposure to COVID-19 (67.4% in Québec vs. 40.5% in Ireland, *p* < 0.001); experienced a separation or divorce during the pandemic (10.1% in Québec vs. 2.62 in Ireland%; *p* = 0.001); could not be accompanied to medical appointments due to COVID-19 (63.7% in Québec vs. 44.2 in Ireland%; *p* < 0.001); and avoided contact with family/ loved ones because of the government recommendations ((92.8% in Québec vs. 78.7% in Ireland;, *p* < 0.001). The COVID-19 stressors with highest intensity of concern or worry from both countries included: losing a job or experiencing a drop in income due to COVID-19; experiencing a separation or divorce during the pandemic; and experiencing the death of a loved one during the pandemic.

### Pandemic and post-pandemic HR-QoL in Ireland and Québec

Table 2 compares overall HR-QoL and specific sub-scales between Ireland and Québec at both timepoints. The mean (SD) overall HR-QoL for all participants was 100.4 (21.6) at T1, while the mean (SD) overall HR-QoL for all participants at T2 was 103.7 (21.2). The mean overall HR-QoL improved significantly from T1 to T2 for the entire sample (*p* < 0.05). The increase was less for Québec compared to Ireland, however the difference was not statistically significant (*p* = 0.66).For both Ireland and Québec, there were statistically significant improvements from T1 to T2 in the PWB, EWB, FWB, and general well-being (FACT-G) subscales (*p* < 0.05), but the differences in improvement between countries were not statistically significant. Participants in Ireland reported significantly lower BCS HR-QoL compared to Québec at both timepoints (*p* < 0.01) but change over time was not statistically different between countries (*p* = 0.95).

**Table 2:** Health-related quality of life (HR-QoL) at timepoint 1 (T1) vs. timepoint 2 (T2).

	Ireland Mean (SD)			Québec Mean (SD)			Ireland vs. Québec t-score		
Health-related quality of life	T1	T2	Change score	T1	T2	Change score	T1	T2	Change score
Overall quality of life (FACT-B)	98.9 (22.3)	102.5 (22.8)	3.67* (15.0)	102.0 (21.0)	104.8 (19.6)	2.96* (14.7)	− 1.31	− 1.02	0.44
Physical well-being sub-scale	21.4 (5.68)	22.3 (5.28)	0.86* (4.58)	21.9 (5.50)	22.7 (5.08)	0.82* (4.69)	− 0.88	− 0.66	0.08
Social well-being sub-scale	19.1 (5.92)	18.8 (6.07)	− 0.30 (4.74)	18.5 (5.12)	18.3 (5.99)	− 0.26 (4.50)	1.00	0.80	− 0.09
Emotional well-being sub-scale	17.0 (4.71)	18.0 (4.51)	1.02* (3.70)	17.8 (4.37)	18.6 (3.86)	0.85* (3.50)	− 1.50	− 1.42	0.43
Functional well-being sub-scale	16.9 (6.46)	18.5 (6.35)	1.58* (5.89)	17.1 (5.90)	18.1 (5.84)	1.10* (5.73)	− 0.39	0.53	0.77
General quality of life (FACT-G)	74.4 (17.2)	77.6 (17.2)	3.36* (11.8)	75.4 (16.5)	77.7 (15.4)	2.54* (12.3)	− 0.52	− 0.05	0.63
Breast cancer specific sub-scale	24.5 (7.08)	24.8 (7.57)	0.31 (5.44)	26.6 (5.93)	27.1 (6.04)	0.35 (4.73)	− 2.99*	− 3.06*	− 0.06

*denotes p-value < 0.05.

### Association between COVID-19 stressor impact and change in HR-QoL by country

Table 3 presents the unadjusted and adjusted associations (adjusting for potential socio-demographic and clinical confounders) between the level of COVID-19 stressor impact and changes in HR-QoL over time. Women with high COVID-19 stressor impact had significantly smaller improvement in overall HR-QoL over time compared to those with low COVID-19 stressor impact, and this was evident in Ireland (*p* = 0.004) and Québec (*p* = 0.001). However, country (Ireland vs. Québec) did not moderate the association between COVID-19 stressor impact and HR-QoL change (*p* = 0.46). Similarly, women from both Ireland and Québec who reported a high COVID-19 stressor impact had significantly smaller improvement in HR-QoL sub-scales, including PWB, EWB, general (FACT-G), and BCS (*p* < 0.05). Women with a high COVID-19 stressor impact experienced a smaller improvement of SWB in Ireland (*p* = 0.001) and a smaller improvement of FWB in Québec (*p* = 0.015). There was no evidence that country significantly moderated the association between COVID-19 stressor impact on these sub-scales (*p* > 0.05).

**Table 3:** Association between COVID-19 stressor impact and change in HR-QoL by country.

	COVID-19 stressor impact High vs. low			
	Unadjusted Coefficient (SE)		Adjusted Coefficient (SE)	
	Ireland	Québec	Ireland	Québec
Overall quality of life (FACT-B) (*n* = 301)	− 10.86* (2.54)	− 10.62* (2.95)	− 7.88* (2.70)	− 11.08* (3.40)
Physical well-being sub-scale (*n* = 309)	− 3.87 (0.71)	− 2.52* (0.82)	− 3.38* (0.79)	− 2.38* (0.98)
Social well-being sub-scale (*n* = 310)	− 3.14* (0.76)	− 1.42 (0.90)	− 2.61* (0.81)	− 0.50 (1.10)
Emotional well-being sub-scale (*n* = 307)	− 1.99* (0.55)	− 1.94* (0.65)	− 1.75* (0.59)	− 2.44* (0.76)
Functional well-being sub-scale (*n* = 306)	− 1.81 (0.91)*	− 3.02* (1.11)	− 1.45 (0.93)	− 2.99* (1.23)
General quality of life (FACT-G)(*n* = 302)	− 8.50* (1.99)	− 8.02* (2.37)	− 6.63* (2.08)	− 8.60* (2.67)
Breast cancer specific sub-scale (n-305)	− 3.16* (0.90)	− 2.94* (1.00)	− 2.21* (1.01)	− 2.66* (1.25)

*denotes p-value < 0.05 between COVID-19 stressor impact and HR-QoL and sub-scales.

SE: standard error.

## Discussion

It is evident from this study that women living with a diagnosis of BC are experiencing on-going impact from the COVID-19 pandemic. In this study, the overall HR-QoL increased to post-pandemic (T2) without significant difference between Ireland and Québec, however HR-QoL did not surpass beyond the minimally important difference (MID) suggested for the FACT-B. To properly interpret changes in HR-QoL over time and group differences for FACT-B, the MID is between 7 and 8 points^30^. When considering the effect of COVID-19 stressor impact on HR-QoL, women with high COVID-19 stressor impact reported significantly smaller improvements in HR-QoL over time for both Ireland (− 7.88 point difference) and Québec (− 11.08 point difference). Therefore, the difference between women who reported a high vs. low COVID-19 stressor impact may be clinically relevant and indicate that COVID-19 stressors may have a long-lasting effect on HR-QoL. Furthermore, the overall HR-QoL post-pandemic for our participants in Ireland was 102.5, and for Québec 104.8, both of which remain sub-optimal compared to existing literature pre-pandemic on similar populations using the FACT-B^31,32^. For example, one study conducted in Canada, published in 2016, found the average FACT-B score among all BC stages, at least two years removed from last treatment, to be 112.6^33^. Consequently, women living with a diagnosis of BC may be experiencing significantly lower HR-QoL post-pandemic.

This is one of the first studies to undertake a cross-country comparison of the association between COVID-19 stressors and the overall well-being of women with BC post-pandemic. The proportion of women with high COVID-19 stressor impact was greater for women in Québec compared to women in Ireland, and there were significant differences in the prevalence of stressors. However, the association between COVID-19 stressor impact and changes in HR-QOL was not moderated by country. In both countries, women who had a high level of COVID-19 stressor impact were significantly more likely to experience a smaller improvement in their HR-QoL, from the pandemic to the post-pandemic period. Adverse COVID-19 stressors and experiences may have exacerbated HR-QoL. A related study conducted during the pandemic found similar COVID-19 stressors to be significantly associated with higher levels of anxiety, depressive symptoms, insomnia, and fear of cancer recurrence^26^. Our study identified the most prevalent stressors to be family/social related (e.g. having a loved one become ill, not being accompanied to medical appointments, and avoiding contact with family and friends) which could be a direct result of restrictive measures on social contacts and gatherings during the pandemic that were implemented and enforced in both Ireland and Québec. Our results indicate that women are still experiencing distress over lived events during the COVID-19 pandemic, therefore, our study illustrates the compounding relationship between living with a diagnosis of BC, the COVID-19 pandemic, and stressful life events on HR-QoL.

### Study strengths

The strengths are that both studies from the two countries were planned and conducted in collaboration. Therefore, eligibility criteria and measures were the same for both settings, and the data were collected at similar timepoints which allows for direct comparisons. Additionally, the study compares two countries with varying contextual factors, health system factors, and COVID-19 pandemic governmental measures which increases the generalizability of results obtained. In addition, the analyses were adjusted for various covariates, including clinical and socio-demographic, that might be associated with HR-QoL.

### Study limitations

The selection of participants into the studies used non-probability sampling, therefore the participants enrolled in the study may not be completely representative to all women living with a diagnosis of BC in both settings. The recruitment strategy also differed in the two settings; Ireland included additional non-probability sampling techniques through cancer centres. Because this sampling technique was not conducted in Québec, there is a potential for differences in the participants’ profiles between the two countries. The current study addressed several potential clinical confounders specific to BC, such as time since most recent diagnosis and cancer stage (e.g. non-metastatic vs. metastatic), however treatment status for BC was not directly included in the analysis. As diagnostic measures and primary treatment for BC during the pandemic were greatly impacted, time since diagnosis includes potential differences in treatment status. Additionally, the study included potential socio-demographic and clinical confounders, however there are likely other behavioural confounders to HR-QoL, such as smoking and physical activity, which were not collected in this study about the COVID-19 pandemic. The study used relatively small sample sizes for some comparisons which may result in under-powered statistical tests. There was some loss to follow-up from T1 to T2, therefore there is a possible risk of bias in interpreting longitudinal HR-QoL. Finally, because the cohort studies were initiated during the COVID-19 pandemic, it was not possible to compare T1 and T2 HR-QoL scores to pre-pandemic HR-QoL scores within our cohort population. However, the results of our study were compared to existing literature on the FACT-B in similar populations and the results were interpreted using clinical scoring (MID).

### Clinical implications

This international comparative longitudinal study found that COVID-19 pandemic stressors may have on-going detrimental effect on the HR-QoL for women living with a diagnosis of BC. BC care should address the on-going impact of the pandemic with appropriate multidisciplinary care to improve sub-optimal HR-QoL, which was evident in both Ireland and Québec, two counties with differing health systems and COVID-19 related contexts. To enhance HR-QoL, psycho-oncology, which is a growing interdisciplinary support for various challenges associated with cancer, can be applied to routine BC care^34^. A successful psycho-oncology intervention should include close-collaboration between psycho-oncologists, medical, and nursing staff and it should also address individual needs as they vary among many factors^35^, including on-going contextual stressors, similar to what was deduced from this research. BC is complex and women living with and beyond a diagnosis BC have varying needs along the cancer continuum. The pandemic may have exacerbated specific needs and disturbed this cancer continuum. BC care teams should be sensitive to the possible on-going impact of pandemic stressors among women who lived with BC during the pandemic.

Those with unmet needs and on-going impacts should be referred to appropriate multidisciplinary care and personalised support services that address various HR-QoL domains, such as PWB, SWB, EWB, FWB, and BCS concerns. COVID-19 stressors should also be addressed in supportive cancer care, like psych-oncology, to enhance overall well-being, especially among women who are continuing to experience high COVID-19 stressor impact. Supportive cancer care should be reinforced during future health crises, in particular to those who are vulnerable, to cope with the combined stressors related to BC and the specific health crisis.

## Conclusion

Women living with a diagnosis of BC were experiencing on-going impact from the COVID-19 pandemic, therefore COVID-19 stressors should be considered as psychosocial determinants of health for this population. While HR-QoL improved from pandemic to post-pandemic, the improvement is still comparatively low to pre-pandemic times. A focus on the HR-QoL of women with BC is necessary moving forward from the COVID-19 pandemic. Identifying and understanding crisis-related stressors is critical as these may have long-term impacts on HR-QoL for women living with a diagnosis of BC. For future health crises, health care professionals should attempt to detect women with high level of stressors and refer them to support services to avoid diminished HR-QoL and these services should be maintained and adapted during future health crises.

## Electronic supplementary material

Below is the link to the electronic supplementary material.

Supplementary Material 1

## Data Availability

The data that support the findings of this study are available on request from the corresponding author. The data are not publicly available due to privacy or ethical restrictions.
