# Supplementary material for: Exploring health related quality of life for women with breast cancer in Ireland and Québec, Canada throughout the COVID-19 pandemic
Source: Sci Rep. 2025 Feb 1;15:4010. doi: 10.1038/s41598-024-84852-9 (PMC11787349; doi:10.1038/s41598-024-84852-9)
Supplement: Supplementary file 1 — Supplementary Material 1 [file 41598_2024_84852_MOESM1_ESM.docx]

Supplementary 1

Prevalence of COVID-19 stressors and intensity of concern by country

|  | Presence of stressor  n (%)^Σ^ | | | Intensity of concern^+^  Median (IQR) | |
| --- | --- | --- | --- | --- | --- |
| Item | Ireland (n=267) | Québec  (n=138) | p-value | Ireland | Québec |
| Became ill following probable or certain exposure to COVID-19 | 82 (30.7%) | 51 (37.2%) | 0.204 | 2 (1-3) | 2 (1-3) |
| Have a loved one become ill following probable or certain exposure to COVID-19 | 108 (40.5%) | 93 (67.4%) | <0.001* | 2 (1-3) | 2 (1-3) |
| Losing a job or experiencing a drop in income due to COVID-19 pandemic | 39 (14.6%) | 15 (11.0%) | 0.275 | 3 (2-3) | 3 (1-4) |
| Having more responsibilities at home due to COVID-19 pandemic | 83 (31.1%) | 41 (29.9%) | 0.793 | 2 (1-3) | 3 (2-3) |
| Having to work in a place likely to expose you to COVID-19 | 77 (28.8%) | 41 (29.7%) | 0.910 | 2 (1-3) | 2 (1-3) |
| Difficulty getting the food, medicine and other essentials you need due to COVID-19 pandemic | 37 (13.9%) | 13 (9.42%) | 0.194 | 1 (1-2) | 2 (2-3) |
| Difficulty getting the support or help you need due to COVID-19 pandemic | 63 (23.6%) | 33 (24.1%) | 0.960 | 2 (1-3) | 2 (2-3) |
| Postponement or cancellation of your cancer treatments due to the COVID-19 pandemic (e.g. delay before starting radiation therapy or postponement of surgery) | 29 (10.9%) | 21 (15.4%) | 0.192 | 2 (1-3) | 3 (2-3) |
| Postponement or cancellation of diagnostic tests and tests of disease progression due to COVID-19 (e.g. blood test, x-ray, magnetic resonance) | 59 (22.1%) | 28 (20.3%) | 0.634 | 2 (1-3) | 3 (2-3) |
| Changes to your cancer treatments as a result of COVID-19 pandemic (e.g. change in the nature of the treatments you normally should have received) | 23 (8.61%) | 13 (9.56%) | 0.779 | 2 (1-3) | 2 (1-3) |
| Experienced a separation or divorce during the COVID-19 pandemic | 7 (2.62%) | 14 (10.1%) | 0.001* | 4 (4-4) | 3 (2-4) |
| Experienced the death of a loved one | 47 (17.6%) | 31 (22.5%) | 0.254 | 3 (2-4) | 3 (2-3) |
| Experienced a change in retirement plans due to the COVID-19 pandemic | 21 (7.87%) | 17 (12.3 %) | 0.152 | 2 (2-3) | 2 (2-3) |
| Experienced conflict with family member or loved ones due to COVID-19 pandemic (e.g. disagreements on health measures, vaccination, etc.) | 74 (27.7%) | 50 (36.5%) | 0.096 | 2 (1-2) | 2 (1-4) |
| Could not be accompanied to your medical appointments due to COVID-19 pandemic. | 118 (44.2%) | 86 (63.7%) | <0.001* | 2 (0-4) | 2 (1-3) |
| Avoided contact with your family/ loved ones who do not live in your home because of the government recommendations | 210 (78.7%) | 128 (92.8%) | <0.001* | 2 (1-3) | 2 (2-3) |

Supplementary 2

Correlations between T1 and T2 HR-QoL scores were high, ranging from 0.61 to 0.78, except for correlations of 0.58 and 0.53 for the FWB sub-scale for Ireland and Québec, respectively. Thus, to compare the change over time between countries, an HR-QoL at T1 by country interaction term was also included in the model. Additional interaction terms with country for the potential confounders were tested for significance using a hierarchical approach, in which potential confounders were strategically entered into the models in stages to test statistical significance.(30) Interaction terms statistically significant for at least one HR-QoL outcome (p<0.05) were included in all models for better adjustment by country. All other variables were included in the model without interaction terms. Adjusted ANCOVA coefficients for COVID-19 stressor impact and their standard errors (SE) are presented. These coefficients can be interpreted as the difference of mean change in HR-QoL over time between high impact group and low impact group.

Coefficient (SE) represents the ANCOVA coefficient and represents the difference in improvement over time of HR-QoL between high and low levels of COVID-19 stressor impact

The unadjusted models include interactions: (T1 HR-QoL x country) and (covid impact x country)

The adjusted models include (T1 HR-QoL x country), (covid impact x country), age, region, employment status, relationship status, cancer stage, and time since diagnosis and the following significant interactions identified via hierarchical approach: (education x country), (income x country) and (co-morbidities x country). Any missing data were omitted from analyses and the data were analysed using Stata Version 16.1
